# Fixation-related potentials during mobile map assisted navigation in the real world: The effect of landmark visualization style

**DOI:** 10.3758/s13414-024-02864-z

**Published:** 2024-03-11

**Authors:** Christopher Hilton, Armand Kapaj, Sara Irina Fabrikant

**Affiliations:** 1 Geographic Information Visualization & Analysis (GIVA), Department of Geography, University of Zurich– Irchel, Winterthurerstr. 190, CH-8057 Zurich, Switzerland; 2 Institute of Psychology and Ergonomics, Technische Universität Berlin, Berlin, Germany

**Keywords:** Spatial navigation, Navigation assistance, Priming, Fixation related potentials, Mobile EEG

## Abstract

An often-proposed enhancement for mobile maps to aid assisted navigation is the presentation of landmark information, yet understanding of the manner in which they should be displayed is limited. In this study, we investigated whether the visualization of landmarks as 3D map symbols with either an abstract or realistic style influenced the subsequent processing of those landmarks during route navigation. We utilized a real-world mobile electroencephalography approach to this question by combining several tools developed to overcome the challenges typically encountered in real-world neuroscience research. We coregistered eye-movement and EEG recordings from 45 participants as they navigated through a real-world environment using a mobile map. Analyses of fixation event-related potentials revealed that the amplitude of the parietal P200 component was enhanced when participants fixated landmarks in the real world that were visualized on the mobile map in a realistic style, and that frontal P200 latencies were prolonged for landmarks depicted in either a realistic or abstract style compared with features of the environment that were not presented on the map, but only for the male participants. In contrast, we did not observe any significant effects of landmark visualization style on visual P1-N1 peaks or the parietal late positive component. Overall, the findings indicate that the cognitive matching process between landmarks seen in the environment and those previously seen on a map is facilitated by more realistic map display, while low-level perceptual processing of landmarks and recall of associated information are unaffected by map visualization style.

## Introduction

### Aided spatial navigation

Spatial navigation is a complex task that humans engage with on a daily basis. As such, navigation aids have become ubiquitous to improve navigation outcomes and minimize disorientation. Modern navigation aids have developed into complex digital tools with design decisions being required for many dimensions. Recent research into design choices has revealed the importance of highlighting landmarks on mobile map displays for efficient navigation and spatial learning (Cheng et al., 2022; Richter & Winter, 2014; Thrash et al., 2019). The present study addresses the next question of whether the visualization style of landmarks on a mobile map display affects subsequent landmark processing via priming of attentional and perceptual processes.

When humans move through space, multiple sensory streams incorporating idiothetic (internal) and allothetic (external) information are processed in their modality specific brain networks before converging in the parietal cortex (Whitlock, 2017) and a wide array of neural systems are recruited to support navigation (Spiers & Barry, 2015). In general, this complex system has been classified as belonging to path integration mechanisms or landmark navigation (Zhao & Warren, 2015). Path integration refers to the self-estimation of movement derived from vestibular and proprioceptive cues, as well as visual optic flow (Etienne & Jeffery, 2004). Landmark navigation refers to the use of external environmental features as cues for action and as stable anchor points for cognitive representations of space (Richter & Winter, 2014). A good landmark has been defined as being stable in the world, unique and visually salient, and situated at a navigationally relevant location (i.e., at intersections; Stankiewicz & Kalia, 2007).

Navigation aids, typically featuring digital and interactive mobile maps, are widely used to relieve the difficulty of navigation by providing information about routes through the environment. The dominant design philosophy for these maps has been to provide minimalist turn-by-turn instructions to the user and visually highlighted routes, overlaid on a 2D footprint of the environment. However, this approach, whilst successful for immediate navigation requirements, has been shown to negatively impact users’ spatial learning and intrinsic navigation abilities with habitual navigation aid use (Dahmani & Bohbot, 2020). It is proposed that the primary explanation for this longitudinal effect is reduced processing of the external world (i.e., reduced engagement of landmark navigation processes) due to the navigation aid device, devoid of environmental information, dominating attentional resources (Gardony et al., 2015). Following given route instructions essentially places the user into a passive navigation state rather than actively engaging with the spatial decision-making process, which is known to reduce spatial learning (Chrastil & Warren, 2012).

To mitigate the negative effect of digital navigation aids, it has been suggested that mobile map designs should feature salient landmark information with the aim of improving landmark-based spatial learning for given routes (Kiefer et al., 2017; Richter & Winter, 2014). Indeed, including references to landmarks in auditory navigation instructions has been shown to induce higher levels of spatial knowledge compared with instructions absent of landmark references, and that the spatial knowledge is longer lasting (Wunderlich et al., 2023). Additionally, the visual presentation of landmarks on a mobile map has also been associated with improved recall of route information (Cheng et al., 2022).

The inclusion of visual landmark information on a mobile map is subject to several design choices. Fully 3D enhanced visualizations of environments, although appealing to users (Hegarty et al., 2012; Lokka et al., 2018), require significant cognitive resources to process and result in a spread of attention across different aspects of the display (Lei et al., 2016; Liao et al., 2017). Indeed, too many highlighted features increases the visual clutter (Rosenholtz et al., 2007) of mobile maps and can induce extra processing demands with a diminished benefit to spatial learning (Cheng et al., 2022; Lokka et al., 2018). For instance, in a lab-based navigation study, Lokka et al. (2018) found that spatial learning performance improved when participants navigated virtual environments (VEs) where only task-relevant landmark buildings were depicted as realistic 3D and the rest of the buildings were depicted as abstract 3D, compared with fully abstract or realistic 3D VEs. It is therefore suggested that enhanced information should pertain to the specific, navigation-relevant landmarks and not necessarily to the whole map (Lei et al., 2016; Liao et al., 2017), which is consistent with the definition of a good landmark being visually distinct from surrounding information. Hence, highlighting only landmarks at navigationally relevant locations as 3D features on a 2D base map is effective at modulating and guiding users’ bottom-up visual attention at these perceptually salient landmark features (Wolfe & Horowitz, 2017). In addition, a realistic 3D visualization offers a more naturalistic representation of the environment (Hegarty et al., 2012), retains the visual properties of landmarks (MacEachren, 1995, p. 259), and facilitates the visual matching process between the information presented on the mobile map display and experienced directly in the environment (Kiefer et al., 2014; Richter & Winter, 2014). Beyond this base, however, there are still many unanswered questions about map design and landmark presentation (Richter & Winter, 2014).

Research into the use of mobile maps with different design decisions is vital to inform best practice and development. In general, human behavioural studies have allowed coarse assessment of spatial memory in response to different stimuli, such as maps with or without landmarks. However, a more fine-grained understanding of the end-user response to navigation aids benefits greatly from investigations into the brain activity of navigators who are guided by mobile maps. Indeed, behavioural responses to different displays can be similar whilst the underlying brain activity shows different patterns, such as higher cognitive load related to increasingly frequent landmark presentation on a map despite a plateau in spatial learning gains (Cheng et al., 2023). Furthermore, not only is understanding neural and attentional engagement important for guided navigation, which almost always takes place alongside other demanding tasks such as driving or walking busy streets, but future research agendas are already set to develop adaptive map technologies (Bartling et al., 2022; Reichenbacher, 2001).

A particularly salient suggestion is the use of neuroadaptive systems that aim to alter the real-time flow of information to the user based on their current state, which must be informed by known neurophysiological signatures and must be grounded in an established methodological paradigm for investigating the design-user relationship (Fabrikant, 2023; Thrash et al., 2019). Taking such investigations out of a restrictive lab environment and into a real-world scenario is important for ecologically valid insights into how design decisions affect the end user, and there is a growing call for more naturalistic approaches to studying the human brain and behaviour (Shamay-Tsoory & Mendelsohn, 2019; Vigliocco et al., 2023). Such a research approach in this field is in its infancy, with only a few studies laying the foundations for the real-world neurophysiological study of navigation aid use (Kapaj et al., 2021; Wunderlich & Gramann, 2021). Therefore, one of the aims of the present study was to extend this approach to investigating mobile map design features. To do so, we contrasted 3D buildings that served as landmarks shown on a planar mobile map that were either displayed with photo-realistic textures (realistic) or without any textures (abstract). This contrast was selected as an example of a fine-grained mobile map decision that cartographers face but that has not yet been empirically assessed, to extend the feasibility of real-world neurophysiological testing.

### Real world neurophysiological testing

Research into spatial navigation behaviour predominantly takes place in a lab environment where participants are seated at a desktop computer, or lay down in a scanner, and input behavioural responses via button press whilst measures such as electroencephalography (EEG) or eye-tracking are simultaneously recorded. Whilst undoubtedly valuable, such approaches restrict some of the natural processes that humans use to navigate, such as path integration. Indeed, studies have already shown that navigation performance is generally better in mobile compared with desktop settings (Montello et al., 2003). Hence, the prevalence of real-world studies to more closely approximate natural navigation behaviour is increasing and has been highlighted as a vital step in the empirical evaluation of navigation aid devices (Fabrikant, 2023). Therefore, in this study, we recorded EEG from actively navigating participants in the real world. Mobile EEG methods yield several challenges compared with traditional desktop methods. Several papers have focused on this contemporary issue (Gramann et al., 2014; Stangl et al., 2023), and in this section we provide a brief overview of those challenges and the respective remedial tools we used to overcome them.

Participant movement during EEG recording gives rise to extensive motion related artifacts which reduce the brain signal-to-noise ratio. These arise from muscle activity and from mechanical movement of cables and devices on the participant. Various procedures have been developed to aid in the processing of these signals, including using independent component analysis (ICA) for the blind separation of signals into statistically maximally independent sources (Bell & Sejnowski, 1995). In the present study, we used the BeMoBIL pipeline (Klug et al., 2022), which incorporates ICA-based cleaning and has been developed to specifically process mobile EEG data.

EEG studies involving a computerized task typically send experimental triggers during recording to be used as event markers in analysis, such as stimulus onset markers, or participant responses. However, when recorded in the real-world in the absence of a computerized task, data streams often contain much fewer event markers, or can lack events entirely. Previous real-world EEG work has relied on the use of eye-movements to create events in the data (Wascher et al., 2014). Wunderlich and Gramann (2021) used blink and saccade events, derived from the EEG ICA components reflecting eye-movements to analyze data recorded from participants who navigated a route whilst receiving navigation instructions. They showed that eye-movement-derived events from mobile EEG can be used to provide insights into the neural activity associated with the contents of navigation instruction and frame their work as a proof-of-concept for this approach. It is noted that a methodological improvement would be the use of a camera-based eye-tracker for better detection of gaze behaviour and would enable more precise fixation-based analyses. Following their advice, in our study, we elected to use eye-movement events for EEG data analysis, derived from a mobile eye tracker, which allowed for eye-fixation-based analysis.

Four key challenges have been identified in the use of coregistered fixations resulting from natural free viewing as events for electrophysiological analyses (Degno et al., 2021; Dimigen & Ehinger, 2021). First, the precise temporal synchronization of independently registered data streams is a vital step to enable cross-data stream event creation since EEG boasts high temporal resolution for examination of neural processes on a millisecond-by-millisecond scale. Our initial approach was to record data using a software platform with multimodal data acquisition solutions. More detail is provided in the Methods section, but to foreshadow, this solution proved inadequate. Post hoc we also used cross correlation of horizontal eye-movement-related ICs in the EEG data and horizontal pupil position of the eye tracking data to optimize the data synchronization (similar to Dimigen & Ehinger’s, 2021, method of cross-correlation between the eye tracker and an electrooculogram signal).

The second challenge is the generation of strong artifacts in EEG data by oculomotor activity. As mentioned earlier, ICA is very effective at separating the signal arising from oculomotor sources which can subsequently be removed from EEG data (Degno et al., 2021; Dimigen et al., 2011). The third challenge is the temporal overlap of evoked brain activity that accompanies fast-paced free-viewing behaviour. The fourth challenge is the existence of low-level covariates that affect the EEG signal, the most notable of which is the effect of the saccade amplitude that precedes a fixation on the early visual perceptual components of the EEG event-related potential (ERP). Ehinger and Dimigen (2019) therefore developed the Unfold Toolbox to address these latter two concerns. This toolbox employs a regression-based separation of overlapping and concurrent brain activity driven by different sources (predictors). Prior studies have successfully used this toolbox to account for overlapping activity associated with blink events (Cheng et al., 2023; Wunderlich & Gramann, 2021) and the effect of prior saccade amplitude on early visual processing (Dimigen & Ehinger, 2021), and to account for the possible association of gait cycles on eye-movement events (e.g., the co-occurrence of blinks and steps in moving subjects in the study by Wunderlich & Gramann, 2021). In the present study, we also utilized the Unfold Toolbox to account for overlapping fixation activity, gait cycles, and the effect of prior saccade amplitude.

### Fixation ERPs

Fixation ERPs are a good solution to create stimulus locked trial-like epochs from continuously recorded unconstrained behaviour. In general, studies have been able to identify typical ERP components from fixation, blink, and saccade-related potentials that have been otherwise largely observed in static laboratory experiments using trial-based stimulus-onset locked epochs. However, there are a few differences with fixation ERP waveforms. Several studies reporting fixation ERPs observe activity in the baseline period. This activity is the saccadic spike potential from the saccade immediately preceding the fixation onset, which is absent in ERPs from experiments that minimize eye movements through participant instruction and by preceding stimuli presented on a screen with fixation crosses.

Fixation ERPs show a large positive component approximately 80–100 ms post fixation onset that is strongest at occipital sites and termed the Lambda response, which replaces the typical visual P1 component (Ries et al., 2018). Investigations into the source of the Lambda response have concluded that it shares the same neural generators as the P1 component and can be interpreted in a similar manner (Kazai & Yagi, 2003). The Lambda response is, as mentioned in the previous section, sensitive to the amplitude of the preceding saccade, with larger amplitudes being seen for larger amplitude preceding saccades (Ries et al., 2018).

The P1 component reflects the processing of information input to the visual cortex and is sensitive to the low-level perceptual aspects of stimuli (Mangun & Hillyard, 1991). It might be expected that prior priming of a landmark via a navigation aid may reduce the perceptual processing demands when fixating on that landmark in the real world; however, the evidence for P1 priming effects is weak. Several studies reported no repetition effects on the visual P1 (Nurdal et al., 2021; Rugg et al., 1995), although other studies have shown some sporadic effects for immediate repetition of objects, but not for delayed repetition (Henson et al., 2004).

Following the initial P1 response, the posterior N1 component occurs ~150 ms post fixation onset and is related to the further visual processing of a stimulus (Wunderlich & Gramann, 2021). It is known to be related to the direction of visual attention (Luck et al., 2000) and the discrimination process of stimulus relevance (Vogel & Luck, 2000). Specifically, lab-based studies have found that the posterior N1 amplitude is enhanced (i.e., more negative) when spatial attention is cued towards a stimulus location prior to onset which coincides with faster discrimination of an object as a target or nontarget (Mangun & Hillyard, 1991; van den Berg et al., 2016). Further still, priming a stimulus through repetition also enhanced the N1 amplitude, but not for objects with an impossible structure (Soldan et al., 2006). Hence, these findings lead to the notion that the posterior N1 is related to the expectancy of a stimulus occurring in a spatial location and integration of an object’s structural features, both of which may benefit from prior exposure to the stimulus. Priming of a landmark in the environment via a navigation aid may be indexed by the N1 component, where visualizations that better prime expectancy and visual familiarity, and focus attention, may result in greater N1 amplitudes when the respective landmark is fixated in the real environment.

Later components past the initial P1-N1 complex reflect higher levels of object processing such as recognition, response associations, or recall processes. Harris et al. (2009) primed participants with line drawings of everyday objects and then tested recognition memory for those objects whilst recording EEG. They reported greater P200 amplitudes over parietal regions for previously seen objects compared with novel objects. Interestingly, they found that this effect occurred independent of explicit recognition accuracy and thus concluded that the P200 enhancement occurs as a result of implicit recognition rather than explicit memory processes. Voss and Paller (2009) also found that previously seen items yielded greater P200 amplitudes in a recognition task than novel items, but in contrast to Harris et al. (2009), they found that explicit recognition memory did modulate the P200. Recognized items classified as ‘known’ were accompanied by greater P200 amplitudes compared with ‘familiar’ responses. The P200 response to familiar items was still higher than new items, but previously seen stimuli classified by participants as a ‘guess’ actually yielded reduced P200 amplitudes compared with novel items. This finding is in line with the notion that the parietal P200 indexes the extent of perceptual matching between the current stimulus and it’s retrieval from memory (Curran & Dien, 2003; Evans & Federmeier, 2007; Voss & Paller, 2009). Hence, navigation aids with higher fidelity landmark visualizations may facilitate the matching processes between the landmark when seen in the real world with the one encoded in memory from the map, which could be visible in the parietal P200 amplitude.

In addition, the P200 over frontal sites has been linked to the allocation of attentional resources, with larger amplitudes indicating a greater engagement of attention (Luck & Hillyard, 1994). In a reading study, participants showed greater P200 amplitudes when fixating target words, which was associated with attentional allocation to task-relevant information, and this effect was enhanced further when participants were provided with more information about the target words via different sensory streams (Boustani et al., 2021). It may be the case that increased information about landmarks on a map display yields better allocation of attentional resources to those features in the environment, due to greater clarity in task-relevancy, which would be reflected in larger frontal P200 amplitudes for realistic landmarks.

Finally, the frontal N400 (FN400) and late positive component (LPC) are widely studied and often co-occurring indicators of familiarity and recall features of memory, respectively (Leynes et al., 2017). Familiarity is often defined as an indicator of a previous encounter with a currently perceived object which supports recognition and later recollection, which is defined as the retrieval of specific details about the object (Yonelinas, 2002). The FN400 component is a negative going deflection over frontal regions occurring between 300 and 500 ms post stimulus that is reduced in amplitude to stimuli encountered before (Curran & Dien, 2003; Leynes et al., 2017). When fixating landmarks that are also visualized on a mobile map, the FN400 may be reduced compared with other environmental features, and more realistic landmark depictions on map displays may further attenuate the FN400 component.

The LPC is a slow-going positivity over parietal sites approximately 500–800 ms post stimulus, which is stronger for known items and is thought to indicate greater information recall (Yang et al., 2019). Previous studies on navigation instruction have found that landmarks included in auditory instructions yield higher LPC amplitudes compared with non-referenced landmarks during simulated (Wunderlich & Gramann, 2018) and real-world navigation (Wunderlich et al., 2023). Hence, if visualization fidelity is important for encoding and retrieval of landmark information, then realistically visualized landmarks should yield higher LPC amplitudes compared with fixations on nonvisualized buildings than abstract landmarks do when fixated in the real-world. Interestingly, this LPC old/new effect is also reduced under divided attention conditions (Curran, 2004), and divided attention is one of the primary explanatory factors in spatial learning deficits associated with navigation aid use (Dahmani & Bohbot, 2020). Therefore, if presentation of realistic landmark symbols serves to reduce the mismatch between map and environment, and thus reduces the requirement to divide attention between the two, then the LPC old/new effect would also be stronger for the fixations on realistic landmark.

### The present study

There were two primary aims in the present study. First, we aimed to assess the effect of different visualization styles of landmark symbols shown on a mobile map on several features of brain activity. Specifically, we investigated how early visual processing, attentional engagement, matching perception and memory, and recollection processes may differ for landmarks previously seen with either an abstract or a realistic visualization style by analyzing the P1, N1, P200, FN400, and LPC components of fixation ERPs. Second, we aimed to do this in a naturalistic real-world context, that more closely approximates real world navigation behaviour and mobile map use compared with a highly controlled lab-based protocol. Hence, we utilized several recent methodological innovations to achieve this goal and in doing so, contribute to the growing body of research converging on a standardized proof-of-concept and pipeline for real-world multimodal research and human-computer interface evaluations.

Participants in this study were required to navigate along a real-world route that was prescribed via a mobile map presented on a tablet device that they carried with them. Relevant landmarks were displayed either as abstract 3D symbols on the mobile map, or as 3D landmark symbols with realistic textures. After the navigation task, participants completed follow up tests of spatial knowledge that are not the subject of this study. That data is presented in a companion article (see Kapaj et al., 2023a), where we found no significant effect of landmark visualization style on landmark recognition, directional recall, sequence knowledge or ability to accurately judge the angles and distances between landmarks in the environment. In the present study, we focused on the neurophysiological activity of participants during active navigation that was guided by a mobile map to assess the in-situ response to different map symbol design decisions.

If perceptual processes occurring with eye fixations on landmarks in the environment were primed by realistic visualizations on the mobile map, then we expected reduced occipital P1 amplitudes for the realistic landmark condition. Conversely, evidence of attentional cueing and stimulus expectancy would be reflected in enhanced occipital N1 amplitudes for the realistically visualized landmarks. If the matching process between the presently fixated landmark and that stored in memory from the map was facilitated, we expected greater parietal P200 amplitudes for fixation ERPs on realistic landmarks, and if the recall of associated information for that landmark was enhanced, then we expected reduced frontal N400 amplitudes and more a more positive deflection in the parietal LPC. Finally, if allocation of attentional resources towards landmarks during navigation was improved by a realistic visualization style on the map, then we expected greater amplitudes in the frontal P200 component when fixating those landmarks in the environment.

## Method

### Participants

We analyzed data from 45 participants (22 females, mean age = 27.32 years; 23 males, mean age = 28.00 years). The sample size was based on the behavioural portion of this study reported in Kapaj et al. (2023a). An a priori power calculation on the primary behaviour measure determined a minimum sample size of 40 (see https://aspredicted.org/6qi6w.pdf), from which we committed to 45 subjects. The study was approved by the Ethics Committee of the University of Zurich, and participants provided written informed consent prior to the start of the experiment and were compensated with CHF 40. Participants reported having normal or corrected to normal vision and no history of any physical or psychiatric disorders.

### Design

**Fig. 1:**
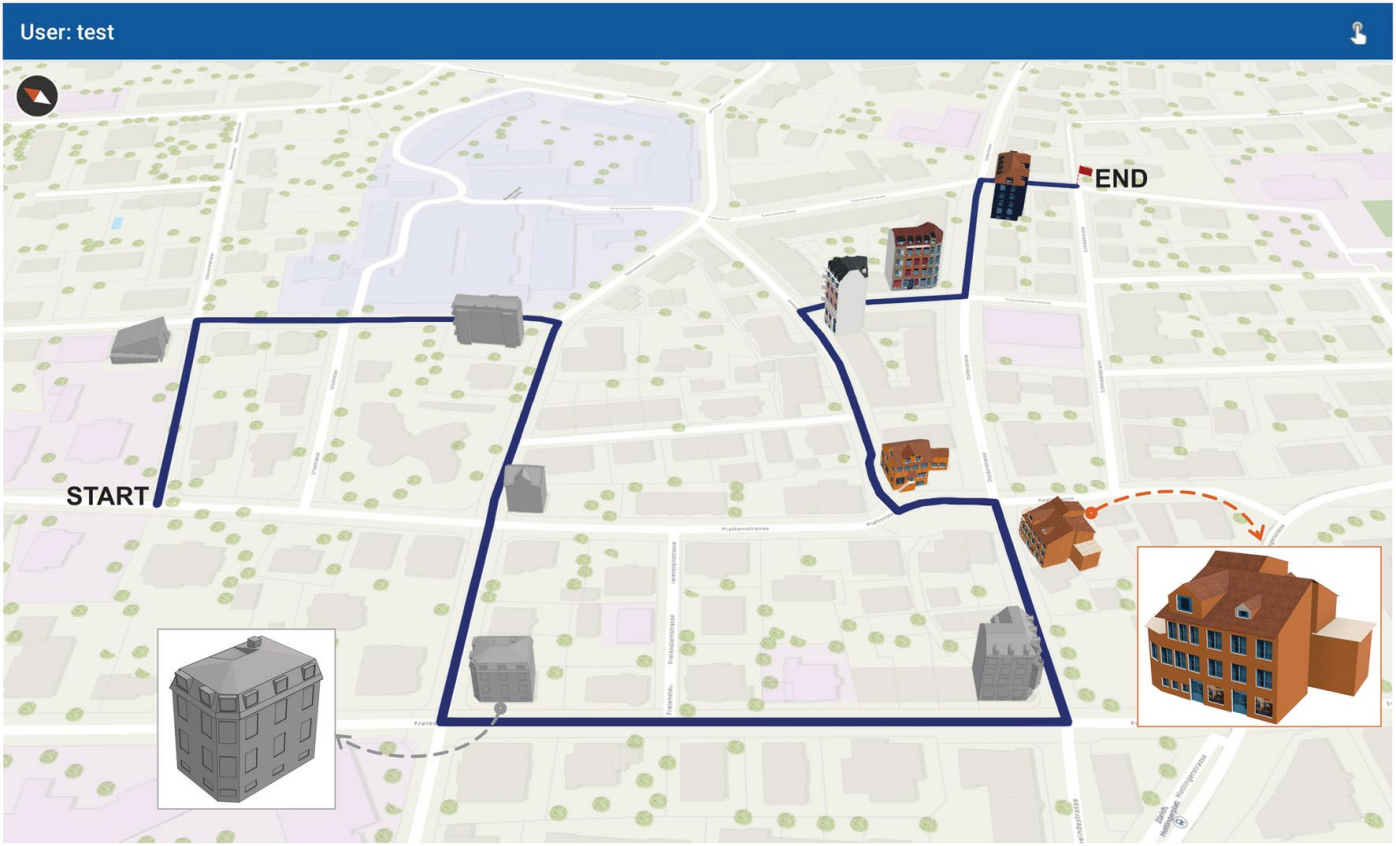
Mobile map display with half of the landmarks depicted as abstract or realistic 3D map symbols. The order of the landmark depictions was counterbalanced across participants, with half of the participants navigating with the first five landmarks depicted as abstract 3D symbols and the other half with the first five landmarks depicted as realistic 3D map symbols. Note that the zoomed-in views and the “start” and “end” labels are for the purpose of this figure and were not present in the experiment. Participants could freely interact with the mobile map, including zooming in/out and rotating the mobile map display. (Colour figure online)

The experiment involved participants navigating a route through a residential neighbourhood in the city of Zurich. The route was prescribed on a mobile map displayed on a tablet device that participants carried with them. The route was approximately 1 km long with five right, four left, and one straight movement at intersections. One building at each intersection was selected to act as a landmark resulting in 10 total landmarks. The landmarks were selected via a survey (*n* = 9) where respondents were shown images of the intersections and were asked to rate the most prominent building that they would use when giving directions and the one that is easiest to describe (cf. Nothegger et al., 2004). The 10 intersection landmarks were featured on the mobile map display as 3D landmark symbols, whilst the rest of the map consisted of 2D outline footprints of buildings (see Fig. 1). The landmark building models were designed in CityEngine 2019.0 (Esri, Redlands, CA, USA), and the mobile map was designed in ArcGIS Pro 2.8 (Esri, Redlands, CA, USA). The stimuli were deployed as interactive mobile map applications using the ArcGIS API for Java (Esri, Redlands, CA, USA) and displayed on a 10.1-in. SAMSUNG Galaxy tablet with a 1,920 × 1,200 resolution. Participants could freely interact—zoom in, zoom out, pan, rotate, and tilt—with the mobile map application. We did not show participants’ location throughout the navigation path, as it has been found to impair spatial learning performance when participants’ offload their self-localization and orientation processes to digital map aids offering positional updates (Brügger et al., 2019).

The experiment was a within-subjects design, with the primary manipulation being the landmark visualization style. Specifically, half the landmarks on the mobile map were shown as abstract nontextured symbols and the other half with realistic textured symbols (see Fig. 1). The order in which the conditions featured was counterbalanced across participants. The main independent variable for this experiment was fixation area of interest (AOI) with three levels. Specifically, fixations were classified as being on a landmark in the external environment that was displayed on the map in a realistic style (henceforth referred to as LM-Realistic), on a landmark that was displayed on the map in an abstract style (henceforth referred to as LM-Abstract), or elsewhere in environment on locations depicted only in 2D on the map (henceforth referred to as Environment).

### Procedure

The experimental session began in a testing room close to the route start site. Here participants received instructions and completed the experiment forms, were given the opportunity to ask questions, and the EEG and eye-tracking devices were set up. Participants were then led to the route start where they were given the digital navigation aid and were instructed to follow the route defined on the displayed mobile map. They had an opportunity to familiarize themselves with the device, and they were instructed to walk as they naturally would when exploring a new environment. The experimenter shadowed participants at a safe distance to record navigation performance and maintain their safety. If participants made an error during the task, they were informed by the experimenter and led back to the previous intersection to continue with the route following task. Participants were asked to raise their hand and indicate whenever they passed a building featured on the map as a landmark as they walked along the route. This was to check that the map was sufficiently representing the environment such that information on the map was applicable to the environment and to ensure that participants engaged with the task. The navigation task lasted approximately 10 minutes. Following the navigation portion of the experiment, participants were guided back to a nearby site to complete several follow-up tasks of spatial knowledge. Those tasks are the subject of another study (Kapaj et al., 2023a) and are beyond the scope of this present study which focuses on active guided navigation behaviour.

### Electroencephalography and eye-tracking measures

#### Acquisition

Eye movements were recorded throughout the navigation task using the Pupil Invisible glasses (Pupil Labs, Berlin, Germany). Eye movements were recorded at 200 Hz, and the tracker featured a front facing scene camera with 1,088 × 1,088 pixel spatial resolution. Clear or shaded lenses were used depending on weather conditions. Data were recorded on an accompanying light-weight mobile device that participants carried in a backpack.

EEG was recorded continuously throughout the navigation task using 64-channel active electrodes (LiveAmp, Brain Products GmbH) arranged according to the 10% system (Chatrian et al., 1985). Data were collected at a 500-Hz sampling rate, band-passed from 0.016 Hz to 250 Hz, and referenced to FCz. We aimed to keep impedances below 10 kOhm. Data were recorded on a laptop, which participants carried in a backpack throughout the experiment, via the iMotions experiment platform (Version 9.1.2, iMotions, Copenhagen, Denmark).

Initial data stream synchronization was conducted using the iMotions multimodal data synchronization tool. This involved an on-screen clock in the iMotions EEG recording interface that participants viewed whilst wearing the eye tracker such that the clock was visible in the scene camera recording. Event markers for the clock were created in the EEG data and then post data collection, eye-tracking data were imported into iMotions and the time stamps of the EEG data and the eye-tracking streams were aligned based on the clock time visible in the scene camera recording. When inspecting the synchronized data, it was clear that this method was not precise and that most datasets had offsets between the EEG and the gaze data that varied substantially between datasets, and we were not able to determine a reliable and systematic offset rule. Therefore, we took additional data processing steps to solve the synchronization issue that are detailed in the following section.

#### Preprocessing

Raw gaze data were manually assigned to AOIs (Environment, LM-Realistic, LM-Abstract) in the iMotions platform using the scene camera recording with overlaid data points. The combined raw EEG and labelled eye-tracking data were then exported from iMotions and data processing continued in MATLAB (R2022b; The MathWorks Inc., 2022) using EEGLAB (Version 2022.1; Delorme & Makeig, 2004). The eye-tracking data were up-sampled to match the EEG data.

EEG data was preprocessed according to the BeMoBIL pipeline (Klug et al., 2022) and plugin for EEGLAB. The single-subject data were low-pass filtered at 124 Hz and were subjected to the ZapLine-Plus function to remove spectral noise using the default automatic detection settings (Klug & Kloosterman, 2022). All datasets had spectral peaks around 50 Hz detected and removed, corresponding to the powerline frequency, and several datasets had peaks around 63 Hz additionally removed. We then used the detect bad channels functions with default settings, including 10 iterations for which channels were rejected if they were detected as bad more than 50% of the time. Rejected channels were interpolated using spherical interpolation in EEGLAB, and the data were rereferenced to the average.

We then used the BeMoBIL wrapper functions to perform Adaptive Mixture Independent Component Analysis (AMICA; Palmer et al., 2011) processing. Prior to AMICA, data were subject to a 1.75-Hz high-pass filter as recommended by Klug and Gramann (2021) for mobile EEG data. We used the default parameters for AMICA including 2,000 iterations and the in-built time domain cleaning option. The high-pass filter and time-domain cleaning were only applied to the data for the purpose of AMICA computations, but not to the final dataset. EEG equivalent dipoles were fit to the resulting ICA decomposition using the dipfit toolbox of EEGLAB with default settings. The computed AMICA data and dipole fitting were copied back to the initial preprocessed dataset without any high-pass filter or data rejected. ICs were labelled using the ICLabel lite classifier (as recommended for mobile EEG data; Klug et al., 2022) using default settings that assigns ICs one of seven labels (“brain”, “eye”, “muscle”, “heart”, “line noise”, “channel noise”, “other”). The final dataset was cleaned with ICA by removing ICs labelled as muscle and eye sources, down sampled to 250 Hz and bandpass filtered between 1 Hz and 30 Hz.

To correct the temporal synchronization of the EEG and eye-tracking data streams we manually identified ICs representing horizontal eye movements in all subjects (prior to ICA cleaning) and correlated this data stream with the horizontal pupil position data from the eye-tracking data. We used a cross-correlation to compute the correlation between the two data streams at different lags to determine the temporal offset present in the data exported from iMotions. When exploring this approach, we noticed that on a few occasions strong correlations emerged for very high lags (e.g., several minutes) that we deemed implausible from examination of the data. Therefore, we restricted the cross correlation to lags ± 10 seconds. The lag with the highest correlation was then applied to the eye-movement data to align it with the EEG data, and we manually inspected each subjects resulting data (average lag = 592.89ms, min = 134 ms, max = 1,776 ms). Overall, this synchronization method worked very well, and this aligned data was taken forward.

#### Event creation, epoching, and unfolding

The eye-tracking data streams were parsed using the EYE-EEG plugin (Version 0.99; Dimigen et al., 2011) for EEGLAB using the adaptive velocity-based algorithm with default settings including a saccade velocity threshold of 6 standard deviations above the individual subject median for at least four consecutive samples (Engbert & Mergenthaler, 2006). Fixation and saccade events were added to the EEG data, from which we excluded very short fixations (<150 ms) and very long fixations (>2,000 ms), and the remaining fixations were assigned an AOI based on the AOI label of the underlying raw gaze samples.

We additionally used the BeMoBIL step detection functions to detect gait related events by manually identifying ICs representing gait cycles in each subjects ICA decomposition. Gait ICs were found for all participants, and in cases where multiple ICs may relate to gait cycles, we used the IC with the highest rank in the decomposition.

Data were subsequently epoched to fixation onset with a −200ms–0ms baseline and separated based on the fixation AOI. There were more fixations in the Environment AOI than in the LM-Realistic and LM-Abstract AOIs (because there were only five of each along the route). The average number of epochs in each condition was: 902.71 Environment, 127.47 LM-Realistic, and 121.44 LM-Abstract.

We used the Unfold Toolbox (Ehinger & Dimigen, 2019) to deconvolute potentially overlapping activity across epochs. In addition to the fixation AOI as a fixed factor, we included the amplitude of the prior saccade (which has been shown to influence the size of the Lambda response; Ries et al., 2018) as a nonlinear predictor as recommended by Dimigen and Ehinger (2021). We also included step events, which prior studies have shown to coincide with blink behaviour in some participants (Wunderlich & Gramann, 2021). Using the single subject coefficients from the resulting unfolded ERP models, we reconstructed the ERP waveforms for analysis.

#### Data analysis

ERPs were computed for occipital, parietal, and frontal sites. Specifically, the occipital ERP was computed using electrodes Oz, O1, and O2; the parietal ERP was computed using Pz, P1, P2, POz, and CPz electrodes; and the frontal ERP was computed using Fz, F1, F2, AFz, and FCz electrodes. Guided by time regions from previous work, we manually identified the components of interest in the grand average waveforms and extracted the latency where each component peak reached maximum amplitude. Using the grand average peak latency ± 30 ms, we searched for individual subject by condition peaks using the findpeaks function in MATLAB, from which we extracted the peak closest to the specified grand average peak latency (cf. Djebbara et al., 2019). For the slower going LPC component we averaged over the component time window. Data were analyzed using linear mixed effects models in RStudio (Version 4.1.1; RStudio Team, 2021) with the lme4 package (Version 1.1-29; Bates et al., 2015) on peak amplitudes and peak latencies with the fixed effect of AOI (treatment contrast coding with environment AOI as baseline), gender (sum contrast coded), and participant ID as a random effect (intercept only). The alpha level was set at 0.05, and any significant interactions were followed up with pairwise comparisons using the EMMEANS package (Lenth, 2024) with Tukey corrections to *p* values for multiple comparisons.

## Results

Navigation performance was overall very high. There were only eight navigation errors out of the 450 total navigation decisions made across five participants—three errors for LM-Abstract intersections made by one participant and five errors for LM-Realistic intersections made by four participants—and only two failures to identify the landmarks along the route made by two participants (one per visualization condition).

**Fig. 2:**
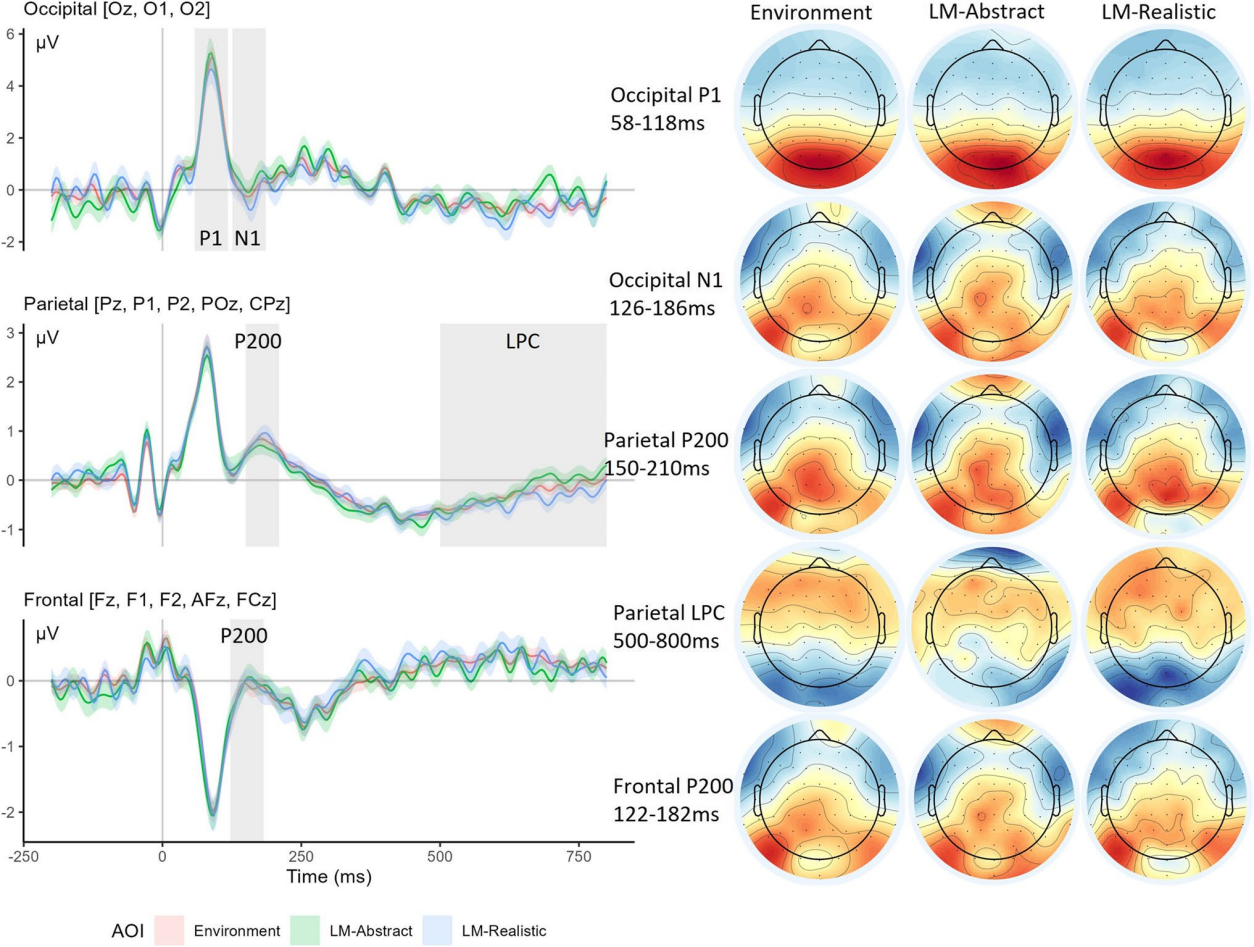
Fixation ERPs for occipital (top), parietal (middle), and frontal (bottom) sensors. Ribbons show standard errors and marked grey regions indicate components targeted for analysis with topographies (right) for their respective time windows. (Colour figure online)

Figure 2 shows the ERPs across occipital, parietal, and frontal sites, and Table 1 reports component peak amplitudes and latencies. The saccadic spike potential from the preceding saccade appears as a negative trough reaching maximum amplitude just before time 0 at posterior and parietal sites. Post fixation onset, a large positive peak occurs at approximately 80 ms, which is the typical Lambda response in fixation ERPs. It is most pronounced at posterior sites as expected, and is volume conducted across all sensors with reducing amplitude towards frontal leads, where the polarity is reversed (cf. Kamienkowski et al., 2012). At approximately 150 ms is the posterior N1 accompanied by a positive going deflection at frontal sites and shortly afterwards also at parietal regions which is consistent with a P200 component. Later in the waveforms a slow-going positive deflection beginning around 500 ms at parietal sites is visible and lasts through to around 800 ms, consistent with the late positive component. Contrary to our analysis plans, there was no evidence of an N400 component at frontal sensors and thus we did not perform any analysis in this time region.

**Table 1:** Mean amplitudes and latencies for each component

	Peak amplitude (µV)			Peak latency (ms)		
	Environment	LM-Abstract	LM-Realistic	Environment	LM-Abstract	LM-Realistic
Occipital P1	6.13	6.78	6.07	91.64	92.82	92.44
Occipital N1	-1.61	-1.59	-2.23	156.20	159.71	158.67
Parietal P200	1.16	1.14	1.44	186.24	185.46	185.27
Parietal LPC*	-0.28	-0.41	-0.19	-	-	-
Frontal P200	0.35	0.62	0.48	151.43	156.27	156.37

*The parietal LPC amplitude is averaged over the 500–800-ms time window

### Occipital region

The *occipital Lambda component* model showed no significant deviation in peak amplitudes from the Environment baseline for the LM-Realistic (β = −0.02, *SE* = 0.32, *t* = -0.07, *p* = .947) or the LM-Abstract (β = 0.57, *SE* = 0.33, *t* = 1.74, *p* = .086) conditions. There was no effect of gender (β = 0.53, *SE* = 0.50, *t* = 1.05, *p* = .296) and no interactions between gender and the LM-Realistic (β = −0.29, *SE* = 0.32, *t* = -0.91, *p* = .366) or LM-Abstract conditions (β = −0.36, *SE* = 0.33, *t* = -1.09, *p* = .279). There were also no significant differences from baseline in peak latencies for the LM-Realistic (β = 0.69, *SE* = 1.67, *t* = 0.42, *p* = .678) or the LM-Abstract (β = 0.92, *SE* = 1.68, *t* = 0.55, *p* = .584) conditions. There was no effect of gender (β = 1.42, *SE* = 1.55, *t* = 0.91, *p* = .364) and no interactions between gender and the LM-Realistic (β = 3.13, *SE* = 1.67, *t* = 1.88, *p* = .064) or LM-Abstract conditions (β = −1.65, *SE* = 1.683, *t* = −0.98, *p* = .329). Note that peaks were not found for one subject in the Environment condition and one subject in the LM-Abstract condition.

The *occipital N1 component* model showed no significant deviation in peak amplitudes from the Environment baseline for the LM-Realistic (β = −0.73, *SE* = 0.44, *t* = −1.64, *p* = .105) or the LM-Abstract (β = −0.09, *SE* = 0.44, *t* = -0.21, *p* = .833) conditions. There was no effect of gender (β = −0.47, *SE* = 0.43,* t* = -1.10, *p* = .275) and no interactions between gender and the LM-Realistic (β = −0.17, *SE* = 0.44, *t* = -0.38, *p* = .703) or LM-Abstract conditions (β = −0.09, *SE* = 0.44, *t* = -0.21, *p* = .832). There were also no significant differences in peak latencies from baseline for the LM-Abstract (β = 2.82, *SE* = 1.91, *t* = 1.48, *p* = .143) or the LM-Realistic (β = 3.82, *SE* = 1.93, *t* = 1.971, *p* = .052) conditions. There was no effect of gender (β = 0.24, *SE* = 2.08, *t* = 0.12, *p* = .909) and no interactions between gender and the LM-Realistic (β = −0.80, *SE* = 1.93, *t* = -0.41, *p* = .681) or LM-Abstract conditions (β = -2.67, *SE* = 1.91, *t* = -1.40, *p* = .166). Note that peaks were not found for four subjects in the Environment condition and three subjects in the LM-Realistic condition.

### Parietal region

The *parietal P200 component* model showed a significantly higher peak amplitude for the LM-Realistic condition than the environment baseline (β = 0.30, *SE* = 0.14, *t* = 2.18, *p* = .033), whereas in contrast, the LM-Abstract condition did not differ significantly from the baseline Environment condition (β = 0.03, *SE* = 0.14, *t* = 0.21, *p* = .835). There was no effect of gender (β = -0.06, *SE* = 0.15, *t* = -0.41, *p* = .683) and no interactions between gender and the LM-Realistic (β = −0.18, *SE* = 0.14, *t* = −1.29, *p* = .201) or LM-Abstract conditions (β = 0.15, *SE* = 0.14, *t* = 1.09, *p* = .278). There were no significant differences from baseline in peak latencies for the LM-Realistic (β = −0.90, *SE* = 2.33, *t* = −0.39, *p* = .700) or the LM-Abstract (β = −0.48, *SE* = 2.34, *t* = −0.20, *p* = .840) conditions. There was no effect of gender (β = 1.51, *SE* = 1.98, *t* = 0.76, *p* = .449) and no interactions between gender and the LM-Realistic (β = −3.44, *SE* = 2.33, *t* = −1.48, *p* = .144) or LM-Abstract conditions (β = −0.78, *SE* = 2.34, *t* = −0.33, *p* = .740). Note that peaks were not found for four subjects in the Environment condition, four subjects in the LM-Realistic condition, and four subjects in the LM-Abstract condition.

The *parietal LPC component* model showed no significant deviation in average amplitude from the Environment baseline for the LM-Realistic (β = −0.13, *SE* = 0.09, *t* = −1.51, *p* = .136) or the LM-Abstract (β = 0.09, *SE* = 0.09, *t* = 1.09, *p* = .280) conditions. There was no effect of gender (β < 0.01, *SE* = 0.08,* t* = 0.08, *p* = .938) and no interactions between gender and LM-Realistic (β = 0.06, *SE* = 0.09, *t* = 0.67, *p* = .507) or LM-Abstract conditions (β = −0.09, *SE* = 0.09, *t* = −1.01, *p* = .316).

### Frontal region

The *frontal P200 component* model showed no significant deviation in peak amplitudes from the Environment baseline for the LM-Realistic (β = 0.12, *SE* = 0.18,* t* = 0.64, *p* = .523) or the LM-Abstract (β = 0.27, *SE* = 0.18, *t* = 1.50,* p* = .138) conditions. There was no effect of gender (β = 0.13, *SE* = 0.18, *t* = 0.75, *p* = .458) and no interactions between gender and LM-Realistic (β = −0.15, *SE* = 0.18, *t* = −0.81, *p* = .421) or LM-Abstract conditions (β = −0.10, *SE* = 0.18, *t* = −0.56, *p* = .576). There were no significant differences from baseline in peak latencies for the LM-Realistic (β = 4.61, *SE* = 2.54, *t* = 1.81, *p* = .074) or the LM-Abstract (β = 4.73, *SE* = 2.53, *t* = 1.87, *p* = .065) conditions. There was no effect of gender (β = 3.79, *SE* = 2.00,* t* = 1.90, *p* = .060) and no interaction between gender and the LM-Abstract (β = −4.31, *SE* = 2.53, *t* = −1.71, *p* = .091) condition. There was a significant interaction between gender and the LM-Realistic condition (β = −6.00, *SE* = 2.54, *t* = −2.36, *p* = .021). We followed up the interaction between gender and condition by performing post-hoc pairwise comparisons between each condition separately for male and female participant groups. There were no significant comparisons for the female participant group (all *p*s > .8), but the male participant group had significantly longer frontal P200 peak latencies in both the LM-Abstract (β = 9.04, *SE* = 3.43, *t* = 2.64, *p* = .027) and LM-Realistic condition (β = 10.61, *SE* = 3.43, *t* = 3.10, *p* = .008) compared with the environment condition. There was no significant difference between the LM-Abstract and LM-Realistic condition for the males (β = −1.57, *SE* = 3.43, *t* = −0.46, *p* = .892). Note that peaks were not found for three subjects in the Environment condition, two subjects in the LM-Realistic condition, and one subject in the LM-Abstract condition.

## Discussion

In this study, participants actively navigated a route through a real-world city neighbourhood using a mobile map to guide their wayfinding. The mobile map displayed navigation-relevant landmarks at intersections depicted in either abstract or realistic fashions. We recorded eye-movement behaviour which was used to classify fixations from real-world free viewing into three AOIs—LM-Realistic, LM-Abstract, and the Environment—that were subsequently used as events for ERP analyses. We found no significant effect of fixation AOI on the occipital P1-N1, parietal LPC component amplitudes or latencies. We found that the parietal P200 component peak amplitude was higher for the LM-Realistic condition, and that the frontal P200 latency was higher for the LM-Abstract and LM-Realistic conditions compared with the baseline environment condition, but only in male participants.

The primary finding was that the P200 component was more positive over parietal leads when fixating realistically visualized landmarks, whereas the amplitude for abstract landmarks did not differ from baseline environment ERPs. The P200 component is related to the perceptual matching process between the currently fixated stimulus and its retrieval from memory, and shows greater amplitudes for previously seen objects compared with novel objects (Curran & Dien, 2003; Evans & Federmeier, 2007; Voss & Paller, 2009). The spatial knowledge tests from this experiment presented in Kapaj et al. (2023a) revealed no differences in landmark recognition performance between the landmark conditions, which is consistent with previous research showing that P200 priming effects can occur in the absence of explicit recognition differences (Harris et al., 2009). Hence, in our study participants had better implicit recognition of landmarks highlighted as important on the mobile map in a realistic style. Increasing environmental learning and strengthening the link between the perceived environment and the environmental information displayed on the map is the primary motivation for visualizing landmarks on mobile maps (Kapaj et al., 2023b; Kiefer et al., 2017; Richter & Winter, 2014). The finding of the present study that improved perceptual matching and implicit recognition of buildings visualized on a map supports the use of landmarks for this goal, and further shows that the landmark visualization style does matter. More realistic visualizations were required to facilitate the matching and recognition process above that of other environmental features.

The frontal P200 latency was prolonged when fixating landmarks visualized in either realistic or abstract styles compared with baseline, but only for the male participants. Amplitude modulations of this component has been linked to the allocation of attentional resources (Allison & Polich, 2008; Ghani et al., 2020). However, we did not observe any effects in this regard. Hence, it appears that males and females did not differ on the extent to which attention was directed towards landmarks, but the speed at which attention was focused on landmarks was actually prolonged in our male participants as a result of being featured prominently on the mobile map. This result somewhat contradicts the parietal P200 benefit for realistic landmark visualizations and is contrary to our expectations, although we highlight that the effect is very small (approximately 5 ms). Males have been shown to have an advantage in some navigation tasks, particularly those involving survey knowledge such as can be gained from studying a map (Castelli et al., 2008). Hence, it is possible that male participants learned more information associated with the landmarks (such as location relative to other landmarks, etc.) from the mobile map which resulted in a delay in attentional resources being allocated to related landmarks encountered later along the route due to a greater maintenance cost. However, this explanation is not supported by our analysis of the LPC, which did not indicate greater information recall for any condition, nor gender. Other work has shown that the frontal P200 latency is prolonged when processing stimuli with a positive afference, and reduced for negative stimuli as a result of attentional prioritization (Carretié et al., 2001). Males generally report greater enjoyment and greater confidence when engaging in spatial tasks than females (Çöltekin et al., 2018; Frenken et al., 2016), and so the extended latency may alternatively be an indicator of task urgency, whereby the female participants prioritize attentional engagement with the task more than males in order to reach the end of a subjectively less enjoyable task.

In contrast to the P200 component, we did not observe any significant differences between conditions on other features of the fixation ERP. Prior studies have shown that the early visual P1 response is not sensitive to priming effects (Nurdal et al., 2021; Rugg et al., 1995), which our study is consistent with and shows that immediate processing of low-level visual features of landmarks are not primed by visualization on a map. Other guided navigation studies have also reported no significant effect of map or instruction manipulation on the P1 component (Cheng et al., 2022, 2023; Wunderlich & Gramann, 2021). The P1 component in ERPs derived from participants with unconstrained eye movements has additionally been shown in another real-world EEG study to index cognitive effort associated with the task (Wascher et al., 2014). Hence, the lack of differences in our study indicates that a similar workload was associated with the early visual perception of all environmental features, regardless of their presentation on a mobile device. In spatial navigation work, Cheng et al. (2023) reported increased P300 amplitudes as an index of cognitive load in accordance with a higher frequency of landmark presentations on a map; however, that study also did not report any differences in the early components of blink ERPs.

We also did not find a significant difference between conditions on the posterior N1 component. This is in line with other studies of guided navigation that utilized eye-movement markers for ERP analyses (Cheng et al., 2022, 2023; Wunderlich & Gramann, 2021). Location expectancy of a stimulus is indexed by the N1 (Mangun & Hillyard, 1991; van den Berg et al., 2016) alongside integration of low-level structural stimulus features (Soldan et al., 2006). Given that both landmark conditions contained structural form-factor information in 3D on the mobile map, and given that all landmarks were visualized at intersections (with counterbalancing between subjects for each half of the route), it is not surprising that the processing of this information did not vary with enhanced visual fidelity. However, the landmark N1 amplitudes did not differ from the baseline environment condition either, which casts doubt on the overall utility of mobile maps for landmark-location priming. Non-assisted navigation studies have shown that adults very efficiently increase the direction of their attentional resources to intersections when navigating (compared with non-intersection portions of a route; Allen & Kirasic, 2003; Hilton et al., 2020). Therefore, it may be that finding landmarks at intersections is not necessarily a feature of spatial learning that requires priming compared with lab-based priming studies that cue screen locations of a randomly appearing stimulus where participants cannot draw upon existing schemas to direct their attention (such as those held for spatial learning; Farzanfar et al., 2023). Furthermore, it has been suggested that disengagement of attention from landmark learning during mobile map-assisted navigation is one of the driving factors behind reduced spatial learning (Gardony et al., 2015). The lack of difference between landmark processing and other nonrelevant features of the environment in our study may indeed be a sign of such disengagement. This hypothesis could be tested in future work by contrasting N1 amplitudes when fixating landmarks during assisted and nonassisted navigation.

Regarding the LPC, we found a prolonged positivity over parietal leads for all conditions from 500–800-ms post fixation onset that is consistent with the LPC (Yang et al., 2019). Wunderlich and Gramann (2018) investigated the effect of landmark type (i.e., relevant or irrelevant) and associated instructions on the LPC amplitude. They found no difference between landmarks presented at intersections (relevant) and irrelevant landmarks. Our results are in line with this, with no difference in LPC amplitude between the baseline environment condition and the landmark conditions. However in contrast to our results, Wunderlich and Gramann (2018) reported that increased information in landmark instructions enhanced LPC amplitude, reflecting an increase in recollection of semantic information associated with the landmarks. In our study, landmark presentation on the mobile maps still required participants to extract associated information such as turning directions and the spatial relations between environmental features, whereas in Wunderlich and Gramann (2018) information was explicitly given via auditory instruction. It could be the case that passive enhancement of map features for readability and increased similarity to the environment is not sufficient to facilitate the derivation of advanced spatial information from the map, such as metric relationships between places or sequences of motor responses. Indeed, the parietal LPC is also reduced under divided attention conditions (Curran, 2004) and thus the lack of an old/new effect between landmarks that were featured on the map compared with the general environment fixations is a sign that the map, in either visualization form, was still dividing attention during navigation.

One of the aims of this study was to utilize coregistered EEG and eye-tracking data from participants actively navigating in the real world to address an applied question about mobile map design and its effect on brain activity. Utilizing the BeMoBIL pipeline (Klug et al., 2022), the Unfold Toolbox (Ehinger & Dimigen, 2019), and eye-movement derived events, our study adds to existing research showing that clear and interpretable EEG signal can be acquired from noisy real-world settings and supports the readiness of current methods for more ecologically valid assessments of human behaviour and brain function (Gramann et al., 2014; Stangl et al., 2023; Vigliocco et al., 2023). Fixations served as effective event markers for ERP analysis where we observed several typical ERP components that have been mostly researched in highly controlled lab settings. One exception to this was the absence of the expected FN400 component. The FN400 amplitude is attenuated by familiar stimuli (Curran & Dien, 2003; Leynes et al., 2017) and one speculative explanation for the absence of this component is that most of the urban neighbourhood may have been somewhat familiar due to high architectural similarity across most environmental features, thus overriding any small familiarity effects that may have been introduced via the navigation aid. This is a consequence of a real-world study where we were not able to control all the features of the traversed environment to minimize visual similarities as can be done in highly controlled lab settings and shows that not all features of the EEG signal are appropriate for hypothesis testing in real-world navigation research.

From a methodological perspective, the temporal synchronization of EEG and eye-tracking data streams was somewhat problematic in our study. To resolve this issue, we utilized a cross-correlation approach to determine and correct the offset between the data streams. Previous work has implemented such a protocol (Dimigen & Ehinger, 2021) using horizontal EOG signals and horizontal pupil position. However, our study lacked EOG recording and therefore we used the horizontal eye-movement component derived from the EEG ICA decomposition instead. This approach was effective and could be a good solution for other work facing similar issues. However, it depends on sufficient ICA quality and manual identification of horizontal eye components, which cannot be guaranteed in real-world mobile EEG and therefore it is best if synchronization of data streams is achieved during recording.

Although the distinction between landmark visualization conditions in our study was subtle, recent work has demonstrated that objects, geometry, and features as cues for spatial learning are separable instances that rely on distinct neural networks (Ramanoël et al., 2022). In a highly controlled fMRI study, Ramanoël et al. (2022) compared objects, geometry (i.e., the structure of the environment such as the buildings used as landmarks in our study), and features (i.e., the colours and textures of walls such as the realistic visualization style in our study) as different landmarks in a navigation task. Some brain regions, such as the hippocampus, were commonly engaged for all three landmark types, but they also observed dedicated neural networks specific to each landmark type. This was complimented by differential patterns of behaviour, including the observation that featural cues lead to more efficient navigation than geometric cues. Ramanoël et al. (2022) pointed out that many studies conflate these different landmark concepts and suggested that a more fine-grained framework of landmark understanding is needed. In our study, we essentially compared the representation of geometric attributes of landmarks alone on a mobile map in our abstract condition and combined geometric and featural attributes in our realistic visualization condition. Although the differences in brain activity between conditions are limited, our study does provide some further confirmation to the notion that the component features of landmarks are important considerations for applied and theoretical spatial navigation research.

One limitation of our study was that ten participants reported having some familiarity with the study area, but that they were not highly familiar. This study was conducted during the COVID-19 pandemic, which presented already great challenges with recruiting participants further away from the study location. This meant that to achieve our desired sample size, we were forced to also recruit participants with some, but modest, familiarity with the traversed study area. To further investigate this limitation, we analyzed the spatial knowledge of those participants and found no effects of familiarity on route knowledge (see companion article; Kapaj et al., 2023a). Additionally, as each participant only navigated the route once, the number of fixation events on each individual landmark was limited. We thus combined all landmarks in this study to achieve sufficient data quantity for clean ERP waveforms. Future work could examine individual features of the traversed urban environment more deeply to understand how different aspects of the built environment may benefit from different levels of representation on mobile map displays.

Taken together, the findings of this pedestrian navigation study show that the visualization style of landmark symbols shown on a mobile map does have some effect on the later neurocognitive processing of landmarks when encountered by navigators in the traversed environment. Specifically, the matching process between the presently fixated landmark in the environment and that which is encoded in memory is facilitated by a more realistic visualization style on the mobile map. However, the recall of information associated with landmarks indexed by the LPC was not enhanced for either of the landmark conditions, suggesting that division of attention and the known detriment to spatial learning and spatial knowledge acquisition induced by navigation aid use (Dahmani & Bohbot, 2020) cannot be attenuated by designing landmark symbols on the mobile map with higher similarity to the landmarks seen in the traversed environment during navigation. In view of other studies showing increased LPC amplitudes from more detailed auditory navigation instructions (Wunderlich & Gramann, 2018), and better implicit landmark recognition from visual enhancements to the landmark map symbols in our real-world study, the solution to navigation aid induced spatial ability decline probably lies in multimodal navigation aids that combine these piecewise improvements (Fabrikant, 2023; Oviatt, 1997). To design such navigation systems, our study shows that real-world multimodal user testing is a viable approach for informing user and environment centred design.

## Data Availability

The datasets analyzed in this study are available from the Open Science Framework open-source online repository: https://osf.io/mzty9.
